# The effect of exercise on the prevention of gestational diabetes in obese and overweight pregnant women: a systematic review and meta-analysis

**DOI:** 10.1186/s13098-019-0470-6

**Published:** 2019-08-27

**Authors:** Fatemeh Nasiri-Amiri, Mahdi Sepidarkish, Marjan Ahmad Shirvani, Payam Habibipour, Narges Sadat Motahari Tabari

**Affiliations:** 1 Fateme Zahra Fertility & Infertility Research Health Center, Health Research Institute, Babol University of Medical Sciences, Babol, P.O.Box: 4717647745, Iran; 2 Department of Biostatistics and Epidemiology, Babol University of Medical Sciences, Ganjafroze Street, Babol, P.O.Box: 4717647745, Iran; 3 Department of Midwifery, School of Nursing & Midwifery, Mazandaran University of Medical Sciences, Vesal Street, Sari, P.O.Box: 4816715793, Iran; 4 Islamic Azad University of Medical Science Sari Branch, Sari, P.O.Box: 194-48164, Iran

**Keywords:** Gestational diabetes mellitus, Exercise, Obese and overweight, Pregnancy, Prevention

## Abstract

**Background:**

Gestational diabetes mellitus (GDM) is one of the most common complications of pregnancy and its prevalence worldwide is increasing along with enhancing type two of diabetes. Contrary results have been found in some review articles that examine the effect of exercise activities on preventing GDM, regardless of obesity. Therefore, the aim of this study was to systematically review the articles on the effect of exercise activities on the prevention of GDM in obese and overweight pregnant women.

**Main text:**

Literature was retrieved by formally searching PubMed, Embase, Cochrane library, Web of Science, Scopus, Proquest and by hand searching of reference lists of related articles. Finally, a total of eight literatures included, and Review manager 5.3 and STATA 14.0 statistical software were utilized for processing. In order to investigate the effect of sports activities on the incidence of GDM, the risk ratio (RR), and for quantitative indices, the standardized mean difference (SMD) with 95% confidence interval (CI) for each study was calculated. Out of 5107 papers identified, eight papers with 1441 participants included in meta-analysis (intervention group 727, control group 714). In the intervention group, 143 (19.66%, 95% CI 76.83 to 22.74) and in the control group, 196 (27.45%, 95% CI 20.24 to 30.88%), pregnant women had diabetes. The RR of gestational diabetes was 0.76 (95% CI 0.56 to 1.03, I^2^ = 50%, P = 0.05). In studies that the time for the intervention was three times a week or less, effect of intervention was significant in reducing the incidence of diabetes (RR: 0.59, 95% CI 0.46 to 0.76, I^2^ = 0%, P = 0.47). However, in studies with repeat of intervention was more than three times a week, the effect of intervention between two intervention and control groups was not different (RR: 1.03, 95% CI 0.78 to 1.35, I^2^ = 0%, P = 0.46).

**Conclusions:**

The exercise activities, alone, in obese or overweight pregnant women did not have a significant effect on the overall incidence of GDM, but considering the effect measure, the incidence of GDM was 24% lower in the intervention group than control group. This difference is considerable in the two groups. As the systematic review literatures both represent the information gap on the research subject and pave the way for further studies so it seems that there is a need for more randomized controlled trials so that we can make a complete conclusion on the type, intensity and duration of exercise in preventing GDM.

## Background

Gestational diabetes mellitus is a disorder of carbohydrate and glucose metabolism, which is first occurring or diagnosed during pregnancy [1, 2]. Due to the physiological, endocrine and metabolic changes during pregnancy in order to meet the nutrient and oxygen requirements of the fetus continuously, the diabetogenic condition similar to that occurring in type 2 diabetes (T2D) is created, increasing the insulin resistance, decreasing the insulin sensitivity and consequently enhancing the need for insulin [3]. In most pregnancies, this need is met and the balance between insulin resistance and secretion is provided. However, if there is no such balance in a person, the symptoms of gestational diabetes are manifested [4]. This disorder is one of the most common complications of pregnancy and its prevalence worldwide is increasing along with enhancing T2D [5].

Gestational diabetes mellitus not only is associated with adverse maternal and perinatal outcomes such as macrosomia, birth weight > 90th percentile for gestational age, increased cesarean section, hypertension, fetal hyperinsulinemia, serum C-peptide level of more than 90th percentile (fetal hyperinsulinemia), preterm labor, shoulder dystocia, birth defects, need for care in the neonatal intensive care unit, hyperbilirubinemia and preeclampsia [5–7], but also increases the risk of long-term problems in mother and infant [8]. Therefore, screening, diagnosis and treatment of GDM are important and necessary to prevent undesirable outcomes.

A number of risk factors affect the incidence and development of GDM. The most common risk factors include obesity and overweight, high maternal age, family history of T2D, previous history of GDM, polycystic ovary syndrome, persistent glucosuria, recurrent abortions, previous history of a large baby (birth weight ≥ 4000 g), history of stillbirth, history of chronic hypertension or blood pressure associated with pregnancy and maternal smoking as well as other risk factors [6, 8]. Among these risk factors, women with overweight, obesity and morbid obesity are related to an increased risk of developing GDM at a rate of two, four and eight times, respectively [9]. With the rise of obesity in the worldwide and the consequent increase in GDM, preventive strategies are needed to avoid the unwanted consequences of obesity and hyperglycemia during pregnancy [10]. Today, the interventions such as lifestyle changes, the use of metformin [11], glyburide [12], myo-inositol [13], insulin [14], diet and exercise activities [15] are applied to prevent and treat the GDM. Contrary results have been found in some review articles that examine the effect of exercise activities on preventing GDM, regardless of obesity [16–18]. A use inexpensive, easy and safe prevention method is preferred in pregnancy. Some studies showed physical activity in pregnancy has these features [19, 20], and also it is effective on insulin resistance [21]. So in the present study, we assessed physical activity from different strategies for preventing diabetes.

Therefore, the researchers of the current study reviewed the articles in which exercise activities were used to prevent GDM in obese or overweight pregnant women. According to the searches, few systematic review articles have been performed related to the effect of exercise activities on GDM in obese and overweight pregnant women, up to now [22].

The studies that have so far been conducted on obese and overweight pregnant women are a combination of lifestyle, diet and exercise on the prevention of GDM, and the independent effect of exercise has not been reported [23–25]. On the other hand, we could find any review about the effect of physical activity on GDM in obese and overweight mothers. Furthermore, since different methods of exercise activities have been used in studies, summarizing the results of these studies can be useful to determine the effective exercise program. Therefore, the aim of this study was to systematically review the articles on the effect of exercise activities on the prevention of GDM in obese and overweight pregnant women in order to achieve a regular summation in this regard.

## Methods

This study was fulfilled through databases and search engines including Medline, Cochrane Library, PubMed, Scopus, Web of Science (WoS), Embase and Cumulative Index to Nursing and Allied Health Literature (CINAHL) databases with interest in studies that reported on the effect of exercise activities on the risk of GDM in obese and overweight pregnant women in the period 1/1/2008 to 5/30/2018 using the relevant keywords. For a complete list of search terms, please refer to Additional file 1. The manual search was also carried out to review the list of references of related articles. In addition, gray literatures were searched in the ProQuest and Prospero, and dissertations and articles presented in conferences were searched in Scopus and WoS. These keywords were selected based on the medical subject headings (MeSH). The advanced search strategy was examined using the operators and tags appropriate to each of the scientific bases.

Inclusion criteria consistent of randomized controlled clinical trials conducted on obese and overweight pregnant women; pregnant women in the control group received routine prenatal care, and the intervention group performed exercise in addition to routine prenatal care. All singleton pregnant women who had no contraindication to exercise. Review and descriptive articles, studies on non-obese and overweight individuals, studies whose interventions were both exercise and other lifestyles such as nutritional modification, studies that did not compare the control and intervention groups and did not answer the research question were excluded from the study.

**Fig. 1:**
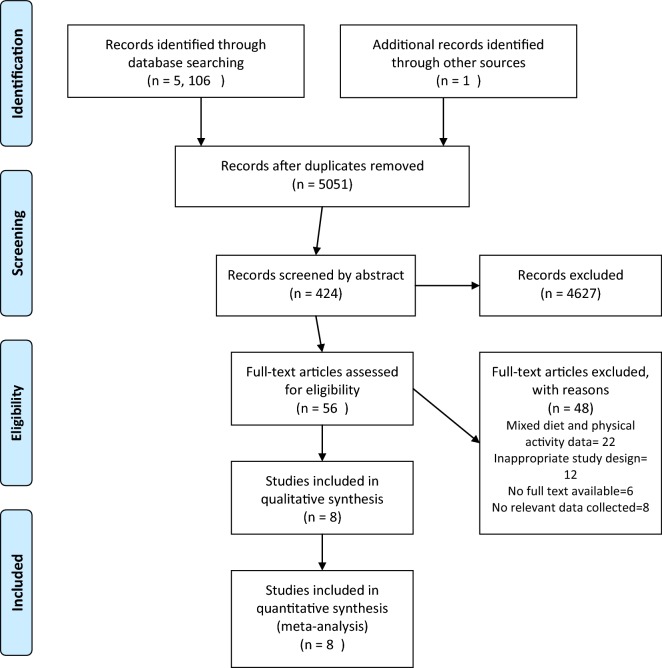
PRISMA flow diagram of screening, selection process and inclusion study

Different stages of study selection and data extraction are shown in Fig. 1. At first, a list of articles was prepared from the databases based on the mentioned keywords. Then, after reviewing the titles of the found articles and removing duplicate titles, two researchers independently reviewed the abstract of articles based on the inclusion and exclusion criteria of the current study. If an article was excluded, the reasons were mentioned. If necessary, the full text of the articles was reviewed. Any disagreement was discussed and if no agreement was found between two researchers, a third researcher independently assessed the article in question. In the next step, the related information of the articles entered into this systematic study including authors’ name, title, year of publication, inclusion and exclusion criteria, sample size, type of intervention, comparison group, outcome and intervention results was recorded. The methodological quality of studies was evaluated according to the recommendation by the Cochrane Handbook, including assessments of the generation of the allocation sequence (selection bias); concealment of the allocation sequence (selection bias); blinding (detection and performance bias); blinding of participants and personnel to outcome assessment; incomplete outcome data (attrition bias); selective outcome reporting (reporting bias); and other bias (https://www.bmj.com/content/343/bmj.d5928.extract).

For each article, the RR was calculated in order to investigate the effect of exercise activities on the incidence of GDM, and standardized mean difference with 95% confidence intervals was calculated for quantitative indices. Data were combined using the random-effects model.

Heterogeneity of the studies was assessed graphically with forest plots and statistically by Chi-square-based Q statistic and I^2^ value. Heterogeneity was considered significant at a P-value of < 0.10 in Q-test or I^2^ > 40%. Subgroup analysis was carried out based on the frequency and time of intervention. Statistical analyses were performed using Review Manager 5.0.1 [26].

## Results

In the initial search, 5107 papers were found in different databases. Totally, 56 articles were selected after screening the titles and abstracts and removing duplicate and unrelated titles. Then, the authors read the full text of the articles and choose eight articles were analyzed (Fig. 1).

The characteristics of the articles studied on the effect of exercise activities on the incidence of GDM in comparison with the control group in overweight and obese women are summarized in Table 1. Different studies were not the same in terms of the type, manner and intensity of exercise. The total number of participants in these studies was 1441 pregnant women. Of these women, 727 and 714 were in the intervention group and control group, respectively. All women were obese or overweight, in the first or second trimester of pregnancy as well as singleton pregnancies without maternal chronic disease and without abnormalities in the fetus. These women may have been nulliparous and multiparous.

**Table 1:** Characteristics of the articles on gestational diabetes in obese and overweight women

Participants							Study characteristic			Out comes			
Author, year, Country	Group No.	Mean age	BMI	Participant	Ges.Age	History	Typ	*D.M-GDM	Setting	Intervention	Neonate	GDM N (%)	OR (CI 95%) P-value
Kirsti Krohn Garnæs (2016) Norway [27]	Int: 46 Con: 45	31.3 ± 3.8 31.4 ± 4.7	33.9 ± 3.8 35.1 ± 4.6	NP* and MP^*^	Baseline 12–18 weeks Late pregnancy 34–37	–	Clinical trial (RCT)	Based on the IADPSG	Trondheim University Hospital	The exercise group was offered thrice weekly supervised sessions of 35 min of moderate intensity endurance exercise and 25 min of strength training Women received Standard Care Controls	–	2 (6.1) 9 (27.3)	0.1 (0.02–0.95) 0.04
Ruben Barakat (2013) Spain [28]	Int: 255 Cont:255	31 ± 3 31 ± 4	24.1 ± 4.1 23.7 ± 3.8	NP and MP	10–12 weeks	–	RCT	WHO criteria and IADPSG	Centro de Los Pedroches and Centro de Salud Leganés Norte, Leganés, Madrid	Moderate-intensity resistance and aerobic exercises (three times/week, 50–55 min/session) Women in control group received the routine care	Apgar score 1 min Apgar score 5 min Birth weight (g) Gestational age (days) Cesarean delivery (n, %)	41 (19.5) 61 (28) 29 (13.8) 32 (14.7)	0.98 (0.40–0.62) 0.040 Based on WHO criteria 0.797 IADPSG criteria
Niamh Daly (2017) Ireland [29]	Int:44 Cont:44	30.0 ± 5.1 29.4 ± 8.4	34.7 ± 4.6 34.7 ± 5.1	NP	less Than 17 weeks	–	RCT	Based on the IADPSG	Coombe Women and Infants University Hospital, Dublin	50–60 min of exercise: warm-up, resistance or weights, aerobic exercises, and cool-down. All women received routine prenatal care	Birth weight (g) Gestation at birth (week) Gestation at birth (week) Apgar scores	25 (58.8) 21 (48.8)	P = 0.51
Oostdam (2012) Netherlands [30]	Int: 59 Cont: 62	30.8 ± 5.2 30.1 ± 5.4	33 ± 3.7 33.9 ± 5.6	NP and MP	After 20 weeks	History of macrosomia OR history of GDM; OR first-grade relative with T2D	RCT	Based on the IADPSG	VU University Medical Center, Amsterdam	The intervention group twice weekly exercises for 60 min Training consisted of aerobic and strength exercises	Gestational age Birthweight, g Caesarean section, % (n) GDM, % (n)	7 (14.6) 11 (21.6)	0.27 (1.55–0.65) 0.37
Chen Wang (2016) China [32]	Int: 133 Cont: 132	32.14 ± 4.57 32.50 ± 4.91	26.82 ± 2.76 26.75 ± 2.75	NP and MP	< 12 + 6 weeks	–	RCT	Based on the IADPSG	Peking University first hospital	The women in the intervention group performed exercises for 50 min three times a week and in a hospital under the supervision of one of the researchers until the end of the week of 37 weeks of pregnancy	Gestational age, Apgar score, birthweight, Apgar score	29 (22) 54 (40.6)	0.412 (0.240–0.705) 0.001
David Simmons (2016) New Zealand, UK, Austria, Poland, Italy, Denmark, Belgium, Netherlands Australia [34]	439 Healthy eating N: 113 Physical activity N: 110 HE&PA: 108 Usual care: 105	Age total: 32.0 ± 5.4	33.7 ± 4.0	NP and MP	< 20 weeks	History of GDM	The DALI Lifestyle Study RCT	Based on the IADPSG	Antenatal clinics a cross 11 centers in 9 European counteries	Interventions start from 20 weeks and up and continue until the 35th week. Both aerobic and resistance physical activity (frequency, intensity, time, type) based on ACOG	Birth weight, gestational age	99 (21.9) 100 (19)	1.21 (0.55–2.67) 0.05 > P
Seneviratn (2016) New Zealand [33]	Int: 37 Cont: 38	–	32.4 ± 4.6 34.5 ± 6.2	NP and MP	From 20 weeks	–	RCT	–	Home-based intervention in Auckland	In this study, individuals in the intervention group performed moderate-intensity home-based exercise programs from week 20 to 35 in moderate intensity three to five times a week, and each time for 15 to 15 min using a steady-state magnetic bicycle	Gestational age (days) Birth weight (g) Occipito-frontal circumference (cm) Ponderal index (g/cm^3^) BMI at birth (kg/m^2^) Placental weight (g) Apgar score Hypoglycaemia Respiratory distress	4 (11) 2 (5)	*2.1* (*0.3–12.8*) P = 0.432
Leonie, Callawa, Fracp Australia [31]	Int: 25 Cont: 25	–	–	NP and MP	12 weeks’ gestation and followed to delivery	–	Pilot randomized controlled trial	Australasian Diabetes in Pregnancy Society criteria were used for the diagnosis and management of GDM	Royal Brisbane and Women’s Hospital	Exercise for the intervention group with the goal of energy consumption up to 900 kcal/week	–	3 (12) 5 (23) 0 (0) 3 (16)	0.07 12 weeks 0.57 28 weeks 0.07 12 weeks 0.57 28 weeks

D.M-GDM*: diagnostic method for GDM; NP*: Nulipara; MP*: multipara

None of the studies has been conducted on women of particular ethnicity or race. A study in Norway [27], a study in Spain [28] a study in Ireland [29] a study in the Netherlands [30], a study in Australia [31], a study in China [32] a study in New Zealand [33] and a multicentre study conducted in nine countries (New Zealand, the United Kingdom, Austria, Poland, Italy, Denmark and Belgium, the Netherlands and Australia) [34] were performed. The methodological quality of the studies entered into the final meta-analysis is illustrated in Table 2. The randomization process was correctly performed in all studies. The randomization and concealment process were not carried out in three studies [29, 33, 34] and explicitly described in two studies [27, 32]. Due to the type of intervention, it was impossible to blind participants in any study. Blinding the outcome measurement was correctly observed in all studies. In five studies, the analytical process was not intention to treat (ITT) [28–30, 33, 34]. The reporting process was in accordance with the protocol. There was only one protocol violation, which was mentioned in the article by the authors [30].

**Table 2:** The methodological quality of the included studies

Author, year	Random sequence generation (selection bias)	Allocation concealment (selection bias)	Blinding of participants and personnel (performance bias)	Blinding of outcome assessment (detection bias)	Incomplete outcome data (attrition bias)	Selective reporting (reporting bias)	Other bias
Callway et al. 2010	−	+	+	−	−	−	−
Oostdam et al. 2012	−	+	+	−	+	−	−
Barakat et al. 2013	−	+	+	−	+	−	−
Seneviratne et al. 2015	−	−	+	−	−	−	−
Simmons et al. 2018	−	−	+	−	−	−	−
Wang et al. 2017	−	?	+	−	+	−	−
Krohn Garnæs1 et al. 2018	−	?	+	−	+	−	−
Daly et al. 2018	−	−	+	−	+	−	−

### Exercise programs

There were different types of exercise program, severity, duration and frequency in studies that included aerobic [29, 30, 32–34], resistance [27, 34], strength [30] exercises and resistance exercises with pelvic floor exercises [29]. In a study, an exercise program with 900 kg calorie per week was conducted through a pregnancy physical activity questionnaire (PPAQ) [31]. In a number of studies, exercise programs began in the first trimester and continued until delivery [31, 32]. In three studies, exercise activities began in the second trimester and lasted until 34–37 weeks of gestation [27, 30, 33]. In two studies, exercise activities started less than 17 weeks (in the first and second trimesters) and continued up to 6 weeks after delivery [29, 34]. In a study, exercise program was repeated twice a week [30]. In four studies, it was repeated three times a week [27, 29, 32, 34]. In a study, the exercise program was daily repeated during a week [31] and repeated three to five times a week in another study [33]. In all studies, the duration of exercise was between 15 and 60 min [27, 29–34].

The intensity level of exercise activities was low to moderate [32, 34] in two studies, moderate to high in three studies [29, 30, 33] and moderate in a study [27], respectively, and it was not determined in one study [31]. In four studies, the intensity scale of exercise activities was based on Borge-Scale [27, 29, 32, 33]. In a study, the intensity level of exercise was according to the American College of Obstetricians and Gynecologists (ACOG) and American College of Sports Medicine (ACSM) guidelines [30]. The ACOG guideline was used to assess the intensity level of exercise in a study [34]. In a study, the intensity level of exercise was at the start of the study based on mother’s maximum heart rate [32]. Another study applied a specific questionnaire for assessing the metabolic equivalent of task to determine the intensity level of exercise [34]. In all studies, the exercise activities were evaluated by an observer [27, 29–32, 34]. In one study, exercise activities were performed only once at home in addition to conducting at the hospital’s clinic [27]. In one study, the exercises were conducted only at home [33].

**Fig. 2:**
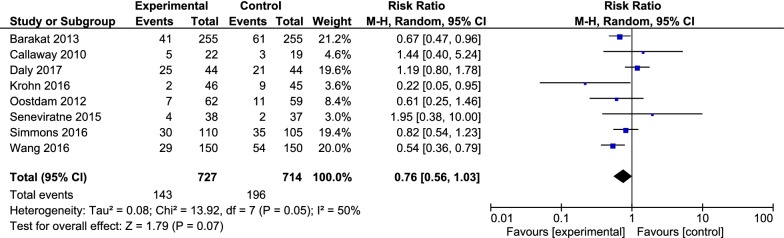
Forest plot of risk ratio of GDM among the intervention and control groups

All included studies of the current research assessed the effect of exercise during pregnancy on a number of pregnancy outcomes. The primary outcome of the recent study was to compare the incidence of gestational diabetes in the intervention group (exercise training during pregnancy) and control group. In the case of diabetes incidence, eight clinical trials with a sample size of 1441 were entered into the final meta-analysis. Accordingly, 143 (19.66%, with 95% CI 16.83 to 22.74) and 196 (27.45%, with a 95% CI 24.20 to 30.88) pregnant women suffered from diabetes in the intervention group and control group, respectively. The RR of GDM was 0.76 (with 95% CI 0.56 to 1.03, P = 0.07). There was moderate heterogeneity between studies (I^2^ = 50%, P = 0.05) (Fig. 2).

**Fig. 3:**
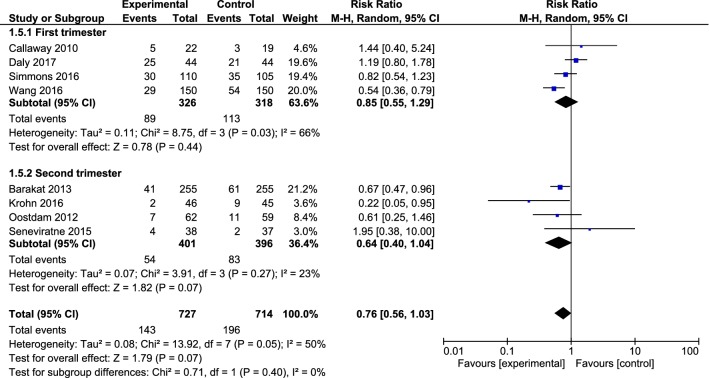
Forest plot of risk ratio of GDM among the intervention and control groups in the first and second trimester of pregnancy

Moreover, there was no statistically significant relationship between the studies with intervention in the first trimester of pregnancy (0.85, with 95% CI 0.55 to 1.29, I^2^ = 66%, P = 0.03) and those with intervention in the second trimester of pregnancy (0.64, with 95% CI 0.40 to 1.04, I^2^ = 23%, P = 0.27) (Fig. 3).

**Fig. 4:**
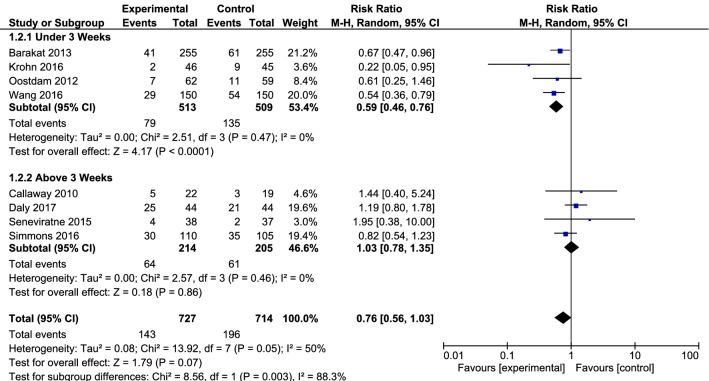
Forest plot of risk ratio of GDM among the intervention and control groups with an intervention time in three times a week or less and an intervention time of more than three times a week

In studies which had an intervention time in three times a week or less, the effect of intervention was observed on reducing the incidence of diabetes (0.59, with 95% CI 0.46 to 0.76, I^2^ = 0%, P = 0.47). However, the effect of intervention on reducing the incidence of diabetes was not seen in studies with an intervention time of more than three times a week (1.03, with 95% CI 0.78 to 1.35, I^2^ = 0%, P = 0.46) (Fig. 4).

**Fig. 5:**
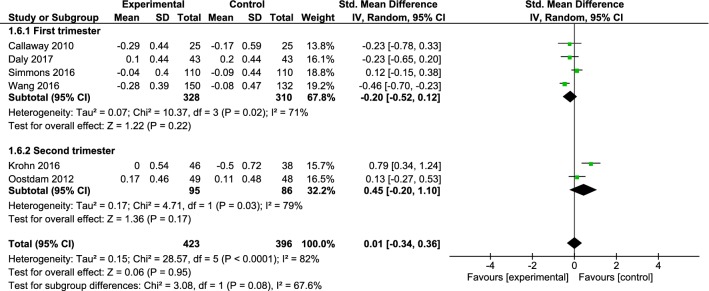
Forest plot of the standardized mean differences FPG the intervention and control groups in the first and second trimester of pregnancy

**Fig. 6:**
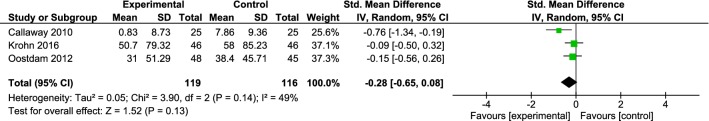
Forest plot of the standardized mean differences FPG among the intervention and control groups with an intervention time in three times a week or less and an intervention time of more than three times a week

Fasting plasma glucose (FPG) was evaluated in six clinical trials with a sample size of 819 participants (423 and 396 in the intervention group and control group, respectively), entered into the final meta-analysis [27, 29–31, 34]. The mean FPG had no significant difference between intervention and control groups (SMD: 0.01, 95% CI − 0.34 to 0.36, I^2^ = 82%, P < 0.001). In addition, no significant difference was found between the studies with intervention in the first trimester of pregnancy [29, 31, 32, 34]. (SMD: − 0.20, 95% CI − 52.0 to 0.12, I^2^ = 71%, P = 0.02) and those with intervention in the second trimester of pregnancy [27, 30] (SMD: 0.45, 95% CI − 0.20 to − 10.1, I^2^ = 79%, P = 0.03) (Fig. 5). There was no significant difference between the intervention and control groups in the studies whose intervention time was three times a week or less (SMD: 0.13, 95% CI − 0.60 to − 0.86, I^2^ = 92%, P < 0.001) like studies with intervention time more than three times a week (SMD: − 0.04, 95% CI − 0.28 to 0.21, I^2^ = 19%, P = 0.29) (Fig. 6).

**Fig. 7:**
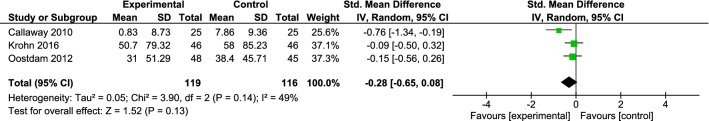
Forest plot of the standardized mean differences FPI among the intervention and control groups

Fasting plasma insulin (FPI) was investigated in three clinical trials with a sample size of 235 participants (119 and 116 in the intervention and control groups, respectively), entered into the final meta-analysis [30, 31, 33]. The mean FPI had no significant difference between two intervention and control groups (SMD: − 0.28, 95% CI − 0.65 to 0.08, I^2^ = 49%, P = 0.14) (Fig. 7).

## Discussion

The objective of this review and meta-analysis study was to determine the effectiveness of exercise activities alone in preventing GDM in obese or overweight pregnant women. The results of the present study showed that the exercise activities, alone, in obese or overweight pregnant women did not have a significant effect on the overall incidence of GDM, but considering the RR, the incidence of GDM was 24% lower in the intervention group than control group. This difference is considerable in the two groups. Furthermore; the effect of intervention on reducing the incidence of GDM was significant in studies whose intervention time was three times a week or less. So that the RR of GDM was up to 41% lower in the intervention group than routine care group. According to the RR, the number needed to treat (NNT) value was 4.2 (confidence ranges from 3.0 to 4.4). The inverse of the absolute risk reduction or increase and the number of patients that need to be treated for one to benefit compared with a control. The ideal NNT is 1, where everyone has improved with treatment and no-one has with control. The higher the NNT, the less effective is the treatment. But the value of an NNT is not just numeric. For instance, NNTs of 2–5 are indicative of effective therapies [35].

Start taking an exercise intervention in the first or second trimesters of pregnancy had no significant difference to decrease the incidence of GDM, but the RR of GDM was up to 36% lower in the intervention group of studies that began the exercise intervention in the second trimester of pregnancy. The review and meta-analysis study of Sanabria-Martínez et al. assessed the effect of physical activity on preventing of GDM and maternal weight gain in 13 studies with 2873 pregnant women. The results of their study also indicated that the risk of developing GDM can be prevented by 31% through physical activities before pregnancy. This prevention of GDM was more evident when exercise was a combination of resistance and aerobic exercises. They have stated that since the resistance exercises lead to blood glucose uptake without varying the muscle capacity to respond to insulin, and aerobic exercises cause glucose uptake via insulin; thus, when these two types of exercise combine with together, the probability of preventing GDM is increased [17].

Du et al. [22] in their review study evaluated the effect of exercise on 1439 pregnant women of 13 studies, and observed that the exercise decreased the risk of GDM in obese and overweight pregnant women. They believed that the heterogeneity in the diagnosis criteria of GDM in various studies may be effective on a result of the study.

However, Han et al. [36] reviewed clinical trials (a total of five articles with 1115 participants) about the impact of exercise on preventing GDM and suggested that there was no significant difference in GDM incidence in women receiving moderate-intensity exercise intervention compared to those receiving routine prenatal care. Moreover, Rogozińska et al. [18] in a review study evaluated the effect of exercise and diet on maternal and fetal outcomes in 24 studies with 8852 participants and found that the exercise, alone, had no significant effect on GDM. In a review study by Yin [16], the effect of physical activity on the risk of developing GDM was assessed in six clinical trials with 1089 pregnant women, and no statistically significant difference was observed in the risk of developing GDM between the intervention and control groups. Besides, Larijani et al. [37] in a review study on GDM women have explained that the upper-body exercises which begin gradually and last 35 to 40 min per day (with two 5-min rest periods during exercise) are as one of the treatments for controlling GDM. This study was conducted not only on obese and overweight pregnant women, but also on all GDM pregnant women. Another study by Khan et al. examined the effects of exercise and diet on maternal outcomes through reviewing 36 articles (with 12,526 pregnant women). After analyzing the results of the studies, the researchers found that observing diet and doing exercise reduce the risk of developing GDM [18]. In the current study, the intervention was a combination of exercise and diet; therefore, the difference between the results of that intervention and those of the present intervention can be due to the differences in intervention type. ‬‬‬‬‬‬‬‬‬‬‬‬‬‬‬‬‬‬

An interesting result of this systematic review study was that doing exercise three times a week or less had better outcomes than doing it more time in preventing GDM, and this difference was statistically significant. This phenomenon may occur through two mechanisms. The stress exerted on the muscles increases the cortisol secretion and cortisol also enhances the blood glucose levels by increasing liver gluconeogenesis and stimulating protein degradation [38]; on the other hand, the body’s metabolism moves into a fat intake and the energy needed to do exercise is obtained by burning fat in people who do daily exercises. As a result, the blood glucose levels of these individuals may remain unchanged or even higher, while when the exercise period is 3 days a week, the body’s metabolism moves to available sources such as blood glucose which reduces the blood glucose [39].

Nasiri and colleagues examined a relationship between the amount of physical activity in the first 20 weeks of pregnancy and the risk of developing GDM in a case–control study. They determined that women with low physical activity in the first 20 weeks of pregnancy, according to the PPAQ questionnaire, were at high risk for development of GDM compared with those who had more physical activity. In addition, after adjusting for age, BMI, gravidity and a family history of diabetes, females with lower physical activity (PPAQ) in the domain of transportation activity during the first 20 weeks of pregnancy were at a significantly higher risk of developing GDM [40].

In this review study, the mean of FBG and FBI changes had no significant difference between intervention and control groups. Motahari et al. studied the effect of eight-week aerobic exercises on insulin resistance in women with T2D. In their study, the participants (who were housewives with T2D) did moderate-intensity aerobic exercise three times a week (daily: 50 min) during 8 weeks. Their results illustrated that the exercise had a significant effect on reducing plasma glucose concentration, insulin resistance and insulin levels, which is inconsistent with the results of the current study [21]. Because their study was conducted on non-pregnant and diabetic women, the exercise had significant effect on the reduction of glucose concentration and, generally, on the control of T2D.

Shakil-ur-Rehman [41] in relation to the effect of exercise on FBG and plasma insulin levels in T2D patients suggested that a 25-day structured aerobic exercise could be a good management of FBG and plasma insulin levels, which are inconsistent with the present study. The study of Shakil-ur-Rehman was also performed on non-pregnant women, which might have resulted in more success of exercise in controlling T2D. In general, these contradictory results represent that more and more precise trials are needed to make a good conclusion.

The researchers of the present study could not investigate the effect of exercise type on the incidence of GDM because of differences in the type of exercise and use of a combination of various exercises in some studies. Besides, the intensity of exercise in all articles of this study was moderate; therefore, it was impossible for the researchers of the current study to assess the effect of different intensities of exercise on the risk of developing GDM. Moreover, the duration of exercise in various studies was between 15 and 50 min, and the lack of access to a sufficient number of studies made it impossible for researchers to compare the exercise duration.

Among the limitations of the current study, the search was only performed in Persian and English, which limited the opportunity to access the trials published in other languages. Unfortunately, due to the lack of studies, there was no possibility to analyze the subgroup for the type and duration of exercise.

The positive aspects of this study were that the HOMA index was used in all studied articles to assess the effects of exercise on insulin level. Furthermore, the intensity of exercise was the same in all early studies (moderate intensity).

Although the effect of exercise on incidence of GDM was not significant, this incidence was considerably lower in the intervention groups. So it seems practitioners may recommend physical activity along with other interventions such as change in life style to prevention of GDM in obese and overweight pregnant women.

## Conclusion

The exercise activities, alone, in obese or overweight pregnant women did not have a significant effect on the overall incidence of GDM, but considering the effect measure, the incidence of GDM was 24% lower in the intervention group than control group. This difference is considerable in the two groups.

Given the above, since the response to exercise in most studies was based on limited evidence and the current research was basically limited to the responses of a hormone to a variety of type, intensity or duration of exercises, and no study was found to consider the various aspects of exercise on other factors affecting gestational diabetes; hence, more trials are needed to actually find the effect of exercise on GDM in obese and overweight pregnant women. As the systematic review literatures both represent the information gap on the research subject and pave the way for further studies so it seems that there is a need for more randomized controlled trials so that we can make a complete conclusion on the type, intensity and duration of exercise in preventing GDM.

## Supplementary information

**Additional file 1.** Searching keywords based on the medical subject headings (MeSH).

## Data Availability

All data generated or analyzed during this study are included in this article.

## Abbreviations

GDM: gestational diabetes mellitus
SMD: standardized mean difference
CI: confidence interval
RR: risk ratio
T2D: type 2 diabetes
WoS: Web of Science
CINAHL: Cumulative Index to Nursing and Allied Health Literature
MeSH: medical subject headings
IADPSG: International Association of Diabetes in Pregnancy Study Group
WHO: World Health Organization
GCT: glucose challenge test
OGTT: oral glucose tolerance test
