# Supplementary material for: The effect of exercise on the prevention of gestational diabetes in obese and overweight pregnant women: a systematic review and meta-analysis
Source: Diabetol Metab Syndr. 2019 Aug 27;11:72. doi: 10.1186/s13098-019-0470-6 (PMC6712661; doi:10.1186/s13098-019-0470-6)
Supplement: Supplementary file 1 — Additional file 1. Searching keywords based on the medical subject headings (MeSH). [file 13098_2019_470_MOESM1_ESM.docx]

**Additional file 1: Searching keywords based on the medical subject headings (MeSH).**

"Obese women" OR "Fat women" OR (Obesity AND women) OR (Women AND Pregnant) OR "Pregnant Woman" AND (Exercises OR "Physical Activity" OR (Activ* AND Physical) OR "Physical Activities" OR (Exercise AND Physical) OR "Physical Exercise" OR "Acute Exercise" OR (Exercise AND Acute) OR (Exercise AND Isometric) OR "Isometric Exercise" OR (Exercise AND Aerobic) OR "Aerobic Exercise" OR "Exercise Training" OR (Training AND Exercise) OR sport) AND (Diabetes AND Pregnancy-Induced) OR (Diabetes AND "Pregnancy Induced") OR "Pregnancy-Induced Diabetes" OR "Gestational Diabetes" OR ("Diabetes Mellitus" AND Gestational) OR "Gestational Diabetes Mellitus") OR Hyperglycemic in pregnancy"
